# Retrospective investigation of the 3D effects of the Carriere Motion 3D appliance using model and cephalometric superimposition

**DOI:** 10.1007/s00784-022-04768-4

**Published:** 2022-11-10

**Authors:** Carmen Ulrike Schmid-Herrmann, Jesper Delfs, Luai Mahaini, Eliane Schumacher, Christian Hirsch, Till Koehne, Bärbel Kahl-Nieke

**Affiliations:** 1 Department of Orthodontics, Center for Dental and Oral Medicine, University Medical Center Hamburg-Eppendorf, Martinistraße 52, 20246 Hamburg, Germany; 2 Orthodontic practice, Laizer Straße 1, 72488 Sigmaringen, Germany; 3 Department of Pediatric Dentistry, University of Leipzig Medical Center, Liebigstraße 12, 04103 Leipzig, Germany; 4 Department of Orthodontics, University of Leipzig Medical Center, Liebigstraße 12, 04103 Leipzig, Germany

**Keywords:** Class II malocclusion, Carriere Motion 3D appliance, Carriere distalizer, 3D evaluation, Model superimposition, Cephalometric superimposition

## Abstract

**Objectives:**

Carriere Motion 3D™ appliance (CMA) represents a method for molar distalization and correction of class II malocclusion. The aim was to investigate the 3D effects of the CMA by superimposing digital models and cephalometric X-rays.

**Materials and methods:**

We retrospectively examined 16 patients treated with CMA in combination with class II elastics. We compared digitized models and cephalometric X-rays of records taken before therapy and after the removal of CMA. The records were superimposed to assess the skeletal and dentoalveolar changes. The results of the cephalometric X-ray analysis were compared to an untreated age- and gender-matched sample.

**Results:**

Class II occlusion was corrected after 11.85 ± 4.70 months by 3.45 ± 2.33 mm. The average distalization of the upper first molars was 0.96 ± 0.80 mm. The analysis of the cephalometric X-rays confirmed a distalization of the upper first molars with distal tipping and revealed a mesialization of the lower first molars of 1.91 ± 1.72 mm. Importantly, CMA resulted in a mild correction of the skeletal class II relationship (ANB: − 0.71 ± 0.77°; Wits: − 1.99 ± 1.74 mm) and a protrusion of the lower incisors (2.94 ± 2.52°). Compared to the untreated control group, there was significant distalization of the upper first molars and canines with mesialization and extrusion of the lower first molars.

**Conclusion and clinical relevance:**

CMA is an efficient method for treating class II malocclusions. However, the class II correction is only partially caused by a distalization of the upper molars.

## Introduction

Class II malocclusion is one of the most common anomalies in orthodontics and affects about one-third of patients [1, 2]. The type of orthodontic therapy depends on the origin of the anomaly. In case of a cause in the lower jaw, functional orthodontic therapy [3] is indicated or, in terms of late treatment, a Herbst appliance [4] or fixed functionals [5, 6] can be applied. However, if the molars in the upper jaw need to be distalized [7], orthodontic headgear [8, 9], Wilson appliance [10, 11], pendulum [12–14], distal slider [15, 16], or distal jet [17] can be used. Other options include class II elastics [18] and the Carriere Motion 3D™ appliance (CMA; Henry Schein Orthodontics, Carlsbad, California), developed by Luis Carrière in 2004 [19].

One advantage of the CMA is its insertion at the beginning of the therapy when compliance is still high [19]. Other advantages of the appliance are the easy insertion and removal, the low invasivity compared to bone-anchored distalization appliances, and the gracile design and size of the appliance compared to the orthodontic headgear.

According to its developer Carrière, indications for the use of the appliance are derotation of mesiorotated upper first molars, straightening of mesially tipped upper first molars, correction of secondary crowding as well as therapy of a class II malocclusion [19].

The ball-and-socket design on the molar pad allows tipping and rotation of the upper first molar. The hook on the canine pad allows class II elastics to the lower first molar, where anchorage is required [19]. In addition, a shorter model is available, which extends from the first bicuspid to the first molar [19]. The bonding protocol of both appliances on the patient is the same. Usually, the normal version (canine to the first molar) is used. If the canine is not erupted (e.g., due to lack of space), the shortened version with attachment to the first premolar can be used.

Finally, the CMA appears to be more comfortable for adolescent patients, offers a positive overall experience, and has fewer negative comfort-related side effects compared to the Forsus™ Fatigue Resistant Device [20].

For the orthodontic treatment, it is of great relevance to what extent the teeth are moved three-dimensionally by the appliance. In fact, as the CMA is often used in combination with class II elastics, it is important to know the dentoalveolar and skeletal effects of the appliance in all three dimensions. However, previous studies have focused on a two-dimensional analysis of the effects of CMA using cephalometric X-rays [21–25]. In addition to the lack of the third dimension, there are overlapping effects of the two halves of the jaw during imaging as well as inaccuracies due to patient positioning in the cephalostat and the X-ray technique. These studies demonstrated that the CMA has a small effect on the skeletal configuration (moderate reduction of the ANB angle and the Wits appraisal) [21–23]. To our knowledge, there are only two studies which analyzed the three-dimensional effects of the CMA [26, 27]. However, these analyses were performed using cone beam computerized tomography, which should not be used for the routine orthodontic patient in order to reduce radiation exposure. In contrast, orthodontic study models and cephalometric X-rays are still regarded as the routine diagnostic procedures. Interestingly, there is no study so far that used superimposition of models to investigate the 3D effects of the CMA. Therefore, the aim of this retrospective study was to evaluate the three-dimensional skeletal and dental effects of the CMA using superimpositions of digitized models and cephalometric X-rays.

## Material and methods

### Subjects

Orthodontic records of 19 cases treated with the CMA were retrospectively evaluated. Due to incomplete records, only 16 cases could be included in this study. The average age at the start of the treatment was 13.9 ± 1.8 years. Seven of the 16 patients were female, and 9 were male. All patients had a class II malocclusion at the start of treatment, on average by 5.25 ± 2.18 mm (evaluated on the models in the region of the 1st molars). Plaster models and cephalometric X-rays of T1 (before therapy) and T2 (after CMA) were available for each case. The characteristics of the study group are shown in Table 1.

**Table 1:** Demographics and characteristics of the study group at the beginning of the treatment

Feature	Value
Patients	*n* = 16
Age	13.9 ± 1.8 years [11.3–17.0 years]
Gender (♂:♀)	9:7
Initial occlusion (U6–L6)	5.25 ± 2.18 mm class II (corresponds to 0.70 ± 0.29 width of a biscupid)
CMA total CMA 3–6 CMA 4–6	32 23 9

For the control group (age and sex controls), the lateral cephalograms of 16 white Northern Americans were derived with permission from the ‘Oregon Growth Study’ and the ‘Denver Growth Study’ through the AAOF Legacy Collection (www.aaoflegacycollection.org).

To enable the comparability among the groups, a correction for linear radiographic magnification was performed for all images, according to the settings of the respective X-ray unit, respectively the instructions provided by the AAOF Legacy Collection. The inclusion criteria for the control group were no history of past orthodontic treatment and the presence of two lateral cephalograms of good quality at similar sex and age to the patients of the treatment group for T1 and T2. Additionally, the controls (*n* = 16) had to have a class II molar relationship.

### Treatment protocol

**Fig. 1:**
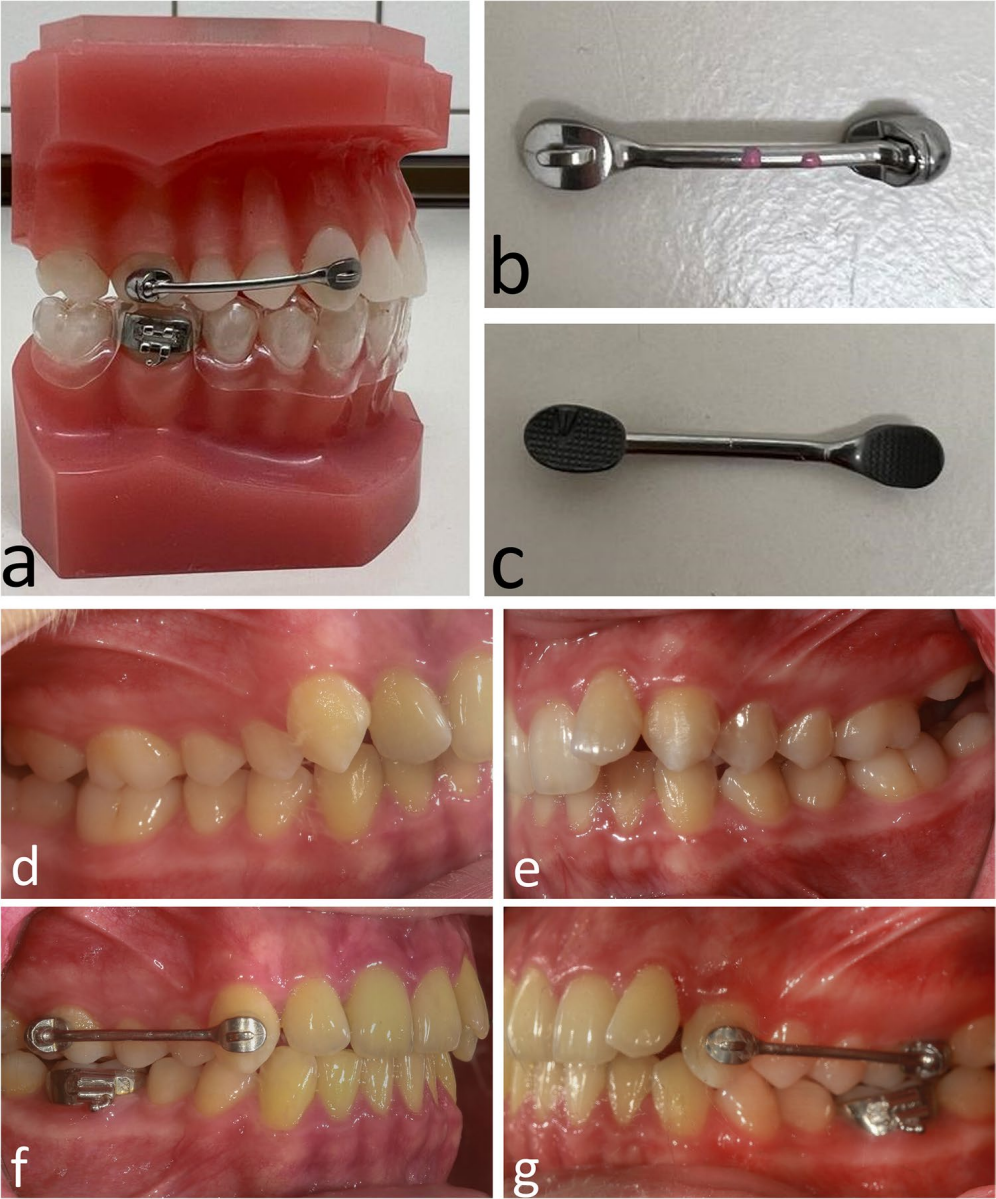
Visualization of CMA on a model (**a**) and before insertion (**b**, **c**). Clinical use of CMA: initial situation (**d**, **e**) and situation after CMA treatment (**f**, **g**)

All 16 patients were treated with CMA (Fig. 1) by the same orthodontist (L.M.). The appliances were used on both sides resulting in a total of 32 appliances (23 appliances from canine to first molar and 9 appliances from first bicuspid to the first molar). Five of the 16 patients had a CMA 3–6 on one side and a CMA 4–6 on the other side. Nine of the 16 patients had a CMA 3–6 on both sides, and 2 of the 16 patients had a CMA 4–6 on both sides. In the mandible, the patients had bands on the first molars and wore an Essix retainer (Duran® 0,5 mm; Scheu Dental, Iserlohn, Germany) extending to the first molars. Patients were instructed to wear class II elastics (initially 3/16” medium/heavy, after 4 months 1/8” heavy) 22 hours a day.

### Model analysis

**Fig. 2:**
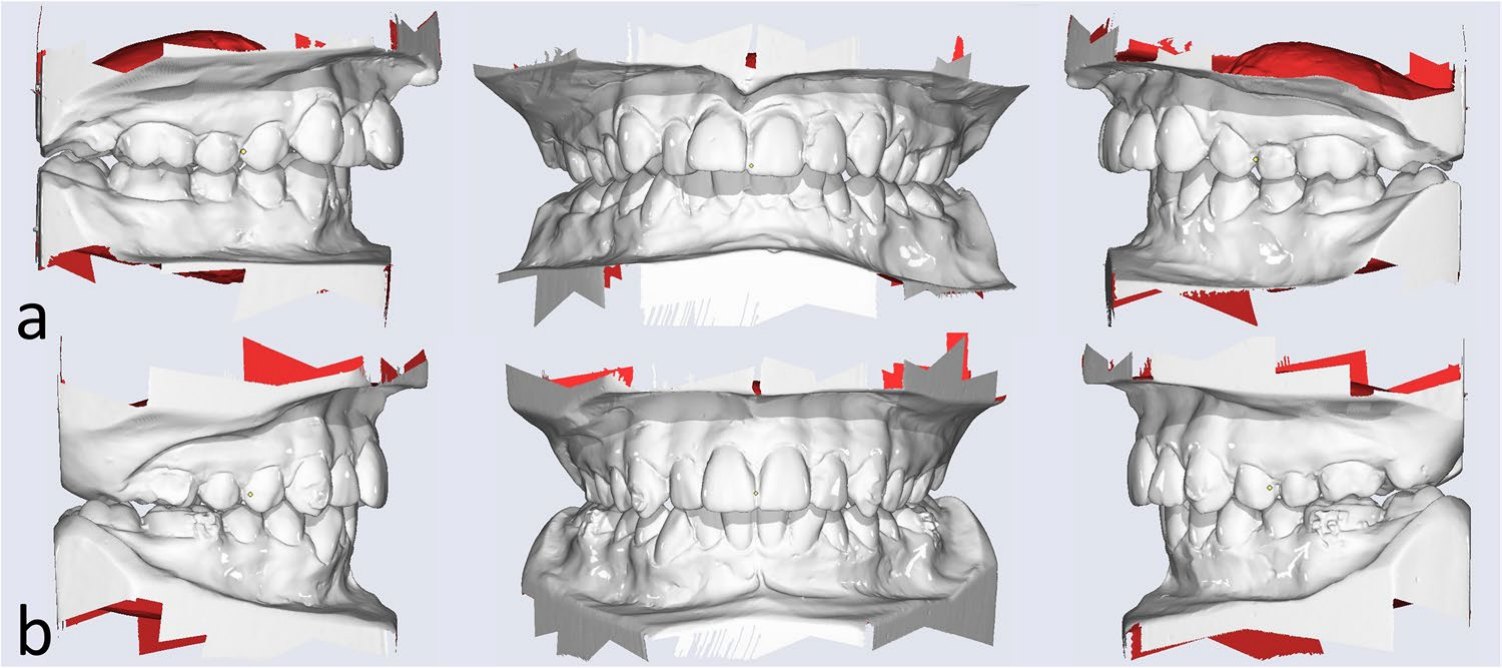
Models digitized with orthoX scan (**a** before and **b** after CMA)

**Fig. 3:**
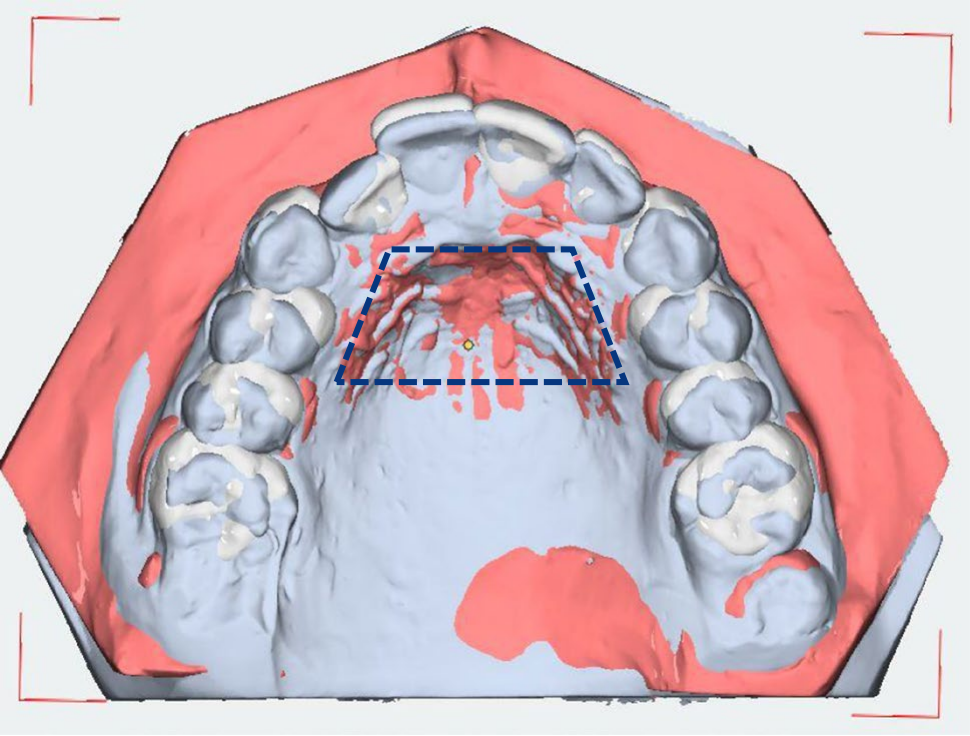
Superimposition of maxillar models (initial and intermediate models) using the palatal folds

The plaster models were scanned with the 3D model scanner orthoX ® scan (Dentaurum, Ispringen, Germany) and imported into OnyxCeph^3^™ (Image Instruments, Chemnitz, Germany; Fig. 2). The models were segmented and superimposed on the palatal rugae [28, 29] to assess the extent of the distalization as well as vertical and transverse changes of the first molars and canines (for CMA 3–6) or first bicuspids (for CMA 4–6) (Fig. 3). The jaws are inserted into a coordinate system as part of the model alignment. By defining the reference points after segmentation, three rotation axes and a vertical axis deviating from the longitudinal axis are created for each tooth in order to quantify both rotational and physical movements.

The amount of class II correction of molars and canines was calculated by evaluating the pre- and posttreatment models. The reference points for molar relationship were the buccal groove of the mandibular first molar and the mesiobuccal cusp of the maxillary first molar. The reference points for the canine relationship were the interproximal contact point between the mandibular canine and the first bicuspid and the cusp of the maxillary canine. The distances between the two points were recorded in millimeters and widths of a biscupid (estimating that an average biscupid corresponds to 7.5 mm) for the left and the right side. The tooth midpoint as well as the tooth axes was calculated using OnyxCeph to determine the three-dimensional changes of the upper first molars and canines/bicuspid. To assess the transverse changes, the anterior and posterior arch width (according to Pont’s index) as well as the intercanine distance was measured in the upper and lower jaw using OnyxCeph.

### Cephalometric analysis

**Fig. 4:**
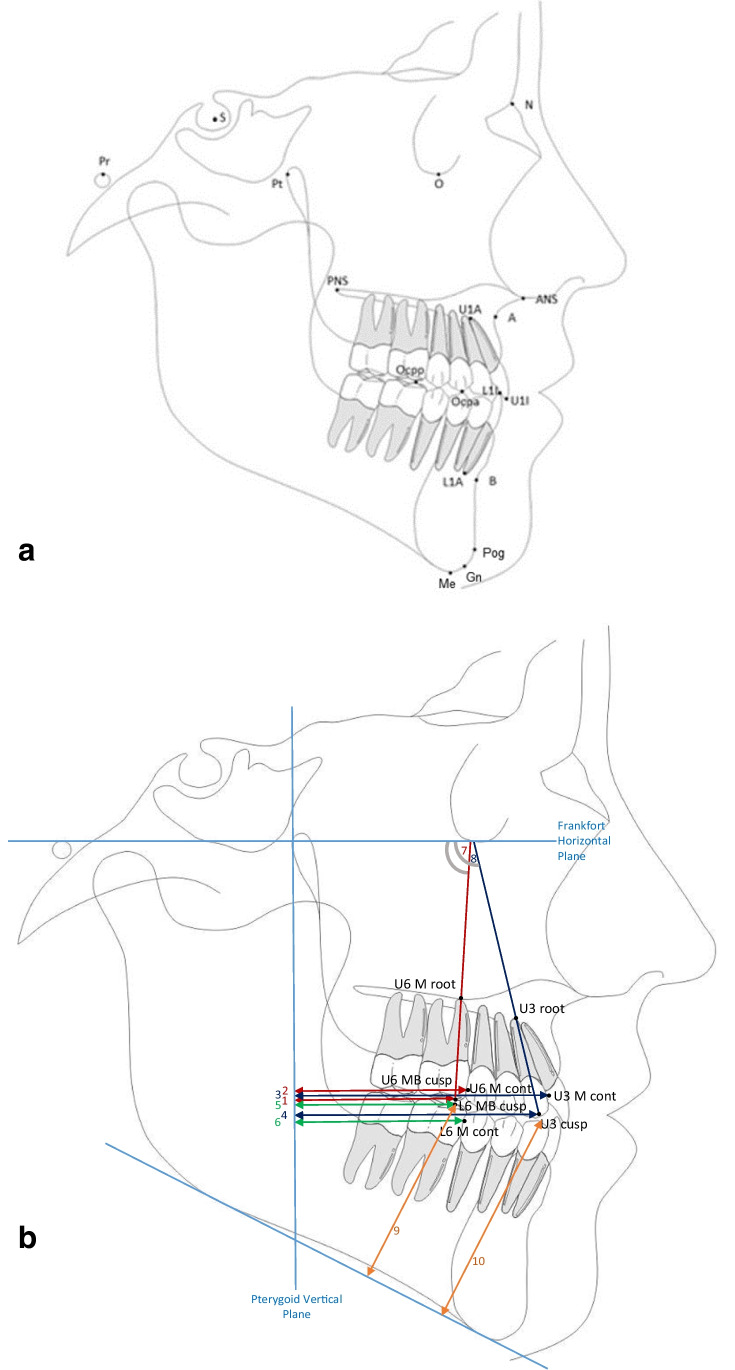
**a** Cephalometric landmarks. **b** Cephalometric analysis according to Kircelli 2006. Linear measurements: (1) U6 MB cusp - PTV, (2) U6 M cont - PTV, (3) U3 cusp - PTV, (4) U3 M cont - PTV, (5) L6 MB cusp - PTV, and (6) L6 M cont - PTV. Angular measurements: (7) U6 – FH and (8) U3 – FH. Additional measurements: (9) L6 MB cusp – GoMe and (10) L3 cusp - GoMe

The cephalometric X-rays (cephalostat: Orthophos 3 Ceph; Sirona, Bensheim, Germany, and PaX-i PCH-2500, Vatech Global, Gyeonggi-do, Korea) were analyzed using ivoris® analyze (Computer konkret AG, Falkenstein, Germany) to assess two-dimensional skeletal and dentoalveolar changes. Twenty-six different cephalometric landmarks were identified (Table 2 and Fig. 4). Bilateral structures were averaged. The cephalograms were traced by two investigators (C.U.S.-H. and J.D.). Changes in cephalometric measurements were calculated as the differences between post- and pretreatment numbers.

**Table 2:** Landmarks, abbreviations, and definitions of the cephalometric analysis

Landmark	Abbreviation	Definition
Subspinale/A point	A	The most posterior midline point in the concavity between the anterior nasal spine and the prothion on the midsagittal plane
Supramentale/B point	B	The most posterior midline point in the concavity of the mandible between the most superior point on the alveolar bone overlying the lower incisors and pogonion on the midsagittal plane
Nasion	N	The most anterior on the frontonasal suture in the midsagittal plane
Sella	S	The geometric center of the pituitary fossa located by visual inspection
Posterior nasal spine	PNS	The posterior spine of the palatine bone constiuting the hard plane
Anterior nasal spine	ANS	The anterior tip of the sharp bony process of the maxilla at the lower margin of the anterior nasal opening
Menton	Me	The most caudal point of the mandibular symphysis
Gonion	Go	The point on the curvature of the angle of the mandible located by bisecting the angle formed by the lines tangent to the posterior ramus and the inferior border of the mandible
Pogonion	Pog	The most anterior point of the bony chin
Gnathion	Gn	The midpoint between the most anterior and inferior point on the bony chin
Porion	Pr	The most cranial point of the porus acusticus externus
Orbitale	O	The most caudal point of the bony orbital contour
Pterygoid point	Pt	The posterior margin of the pterygomaxillary fissure
Posterior point of the occlusal plane	Ocpp	The posterior point of the occlusal plane (mesiobuccal cusp tip of the mandibular first molar)
Anterior point of the occlusal plane	Ocpa	The anterior point of the occlusal plane (cusp tip of the mandibular first bicuspid)
Incision superius	U1I	The incisal tip of the maxillary incisor
Apex OK1	U1A	The tip of the root of the maxillary incisor
Incision inferius	L1I	The incisal tip of the mandibular incisor
Apex UK1	L1A	The tip of the root of the mandibular incisor
Maxillary molar mesiobuccal cusp tip	U6 MB cusp	The mesiobuccal cusp tip of the maxillary first molar
Maxillary molar mesial approximal contact	U6 M cont	The mesial contact of the maxillary first molar
Maxillary molar mesial root apex	U6 M root	The mesiobuccal root apex of the maxillary first molar
Maxillary canine cusp tip	U3 cusp	The cusp tip of the maxillary canine
Maxillary canine mesial approximal contact	U3 M cont	The mesial contact of the maxillary canine
Maxillary canine root apex	U3 root	The root apex of the maxillary canine
Mandibular molar mesiobuccal cusp tip	L6 MB cusp	The mesiobuccal cusp tip of the mandibular first molar
Mandibular molar mesial approximal contact	L6 M cont	The mesial contact of the mandibular first molar

The cephalometric tracings were used to determine the sagittal position of the jaws (SNA, SNB), the skeletal class (ANB, Wits Appraisal, individual ANB), the growth pattern (*Y*-axis angle), and the anterior tooth inclination in the upper and lower jaw (U1-SN, U1-SpE, L1-MeGo).

The pterygoid vertical (orthogonal to the Frankfort horizontal through Pt) was constructed according to the analysis of Kircelli et al. [30]. These measurements included the distance of the mesiobuccal cusp tip of the upper first molar, the mesial contact point of the upper first molar, the cusp tip of the upper canine, the mesial contact point of the upper canine, the mesiobuccal cusp tip of the lower first molar, and the mesial contact point of the lower first molar to the pterygoid vertical. In addition, we determined the inclination of the upper first molar (tooth axis: mesiobuccal cusp tip to the mesial root tip) and the upper canine (tooth axis: cusp tip to root tip) to the Frankfort horizontal plane (Table 3, Fig. 4).

**Table 3:** Abbreviations and definitions of the cephalometric measurements

Abbreviation	Definition
SNA (°)	The angle between Sella-Nasion-Subspinale
SNB (°)	The angle between Sella-Nasion-Supramentale
ANB (°)	The angle between Subspinale-Nasion-Supramentale
Wits appraisal (mm)	The distance between the orthogonal of the A point and the orthogonal of the B point to the occlusal plane
Individual ANB (°)	− 35.16 + 0.4*(SNA) + 0.2*(SN-MeGo)
NS-Gn (*Y*-axis; °)	The angle between Sella-Nasion-Gnathion
Occlusal plane - NS (°)	The angel between the Nasion-Sella-occlusal plane
U1 - NS (°)	The angle between the long axis of the upper incisor (cusp tip to root apex) to the NS plane (anterior skull base)
U1 - SpE (°)	The angle between the long axis of the upper incisor (cusp tip to root apex) to the spina plane (ANS-PNS)
L1 - GoMe (°)	The angle between the long axis of the lower incisor (cusp tip to root apex) to the mandibular plane (GoMe)
U6 MB cusp - PTV (mm)	The horizontal distance from the mesiobuccal cusp tip of the maxillary first molar to the Pterygoid vertical
U6 M cont - PTV (mm)	The horizontal distance from the mesial contact of the maxillary first molar to the Pterygoid vertical
U6 axis - FH (°)	The angle between the axis of the maxillary first molar (mesiobuccal cusp tip to mesiobuccal root apex) to the Frankfort horizontal plane
U3 cusp - PTV (mm)	The horizontal distance from the cusp tip of the maxillary canine to the Pterygoid vertical
U3 M cont - PTV (mm)	The horizontal distance from the mesial contact of the canine to the Pterygoid vertical
U3 axis - FH (°)	The angle between the axis of the maxillary canine (cusp tip to root apex) to the Frankfort horizontal plane
L6 MB cusp - PTV (mm)	The horizontal distance from the mesiobuccal cusp tip of the mandibular first molar to the Pterygoid vertical
L6 M cont - PTV (mm)	The horizontal distance from the mesial contact of the mandibular first molar to the Pterygoid vertical
L3 - GoMe (mm)	The vertical distance from the cusp tip of the mandibular canine to the mandibular plane (GoMe)
L6 - GoMe (mm)	The vertical distance from the mesiobuccal cusp tip of the mandibular first molar to the mandibular plane (GoMe)

### Statistic analysis

For our sample size calculation, we used the pilot study of Yin et al. (2019) and their sample size calculation (minimum required sample size: *n* = 16). Furthermore, we used a G-Power analysis (https://www.psychologie.hhu.de/arbeitsgruppen/allgemeine-psychologie-und-arbeitspsychologie/gpower) to estimate the sample size.

The statistical evaluation was performed using Excel (Excel 2013, Microsoft, USA) and GraphPad Prism (GraphPad Software, USA). All data were normally distributed. The mean numbers and standard deviations are reported for descriptive statistics. Significant differences between cephalometric variables of the CMA patients were determined using a paired two-sided *t*-test. Significant differences between the mean differences of the cephalometric variables between CMA and control patients were determined using an unpaired two-tailed *t*-test (*α* level = 0.05). A sample *t*-test was used to test whether the mean differences of the model measurements were significantly different from 0.

The model analysis and cephalometric analysis were performed by two investigators: C.U.S.-H. and J.D. The reliability of repeated measures was calculated using intraclass correlation coefficients (ICC, SPSS V.28, IBM SPSS Statistics, USA).

## Results

**Fig. 5:**
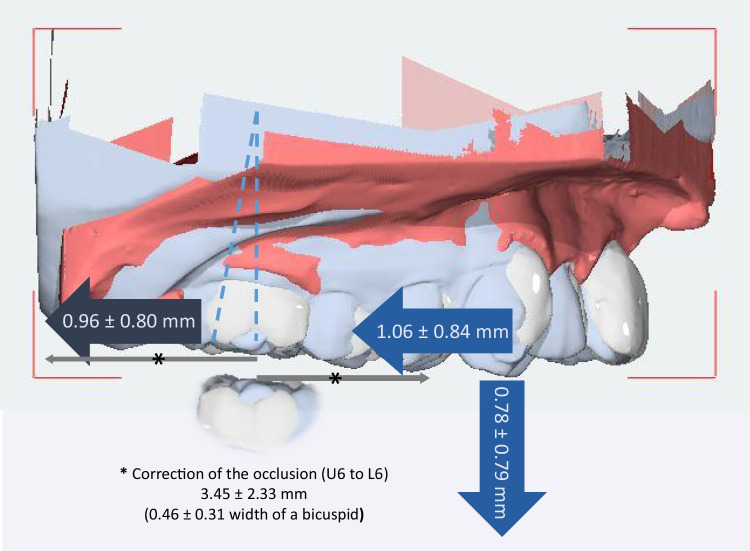
3D effects with distances (including standard deviation) of the CMA using the model superimposition (gray small arrows: occlusal correction; blue horizontal arrows: pure distalization; blue vertical arrow: extrusion)

The average wear time of the CMA was 11.85 ± 4.70 months. The occlusion changed by 3.45 ± 2.33 mm (0.46 ± 0.31 width of a biscupid; measured between the buccal groove of the mandibular first molar and the mesiobuccal cusp of the maxillary first molar; Fig. 5).

### Statistic results

Assuming a moderate effect (*d* = 0.7) and an alpha error of 0.05, the sample size calculation using the G-Power analysis yields a total sample size of *n* = 13.

Intraclass correlation coefficients (ICC) showed good interindividual and intraindividual agreement (interindividual: mean ICC: 0.89, range: 0.84–0.97; intraindividual: mean ICC: 0.91, range: 0.86–0.96).

### Model results

The results of the measurements using the model superimposition at T1 and T2 are described in Table 4.

**Table 4:** Results of the measurements of the model superimposition

Direction	U3 (CMA 3-6)	±	U4 (CMA 4-6)	±	U6	±
+: mesialization -: distalization (mm)	− 1.10***	0.89	− 0.94**	0.72	− 0.96***	0.80
+: palatinalization -: buccalization (mm)	− 0.40**	0.64	0.12	0.53	− 0.53**	0.68
+: extrusion -: intrusion (mm)	0.82***	0.87	0.66**	0.54	0.03	0.77

**p < 0.01, ***p < 0.001

The average distalization of the first molars was 0.96 ± 0.80 mm. The vertical position of the molars was not changed (0.03 ± 0.77 mm). At the level of canines/first bicuspids, we observed an average distalization of 1.10 ± 0.89 mm (canines)/0.94 ± 0.72 mm (first bicuspids) and an average extrusion of 0.82 ± 0.87 mm (canines)/0.66 ± 0.54 mm (first bicuspids).

Furthermore, we detected a small movement of the canine and the first molar in the buccal direction (canines: 0.40 ± 0.64 mm; first molars: 0.53 ± 0.68 mm).

The distalization of the first molars, canines, and first bicuspids was significant. The canines and first molars showed a significant buccal movement, while the canines and first premolars showed a significant extrusion.

The results of the transverse changes are described in Table 5.

**Table 5:** Results of the transverse model measurements

Measurement	Mean value	±
Posterior arch width upper jaw (mm)	− 0.24	1.10
Anterior arch width upper jaw (mm)	0.69	1.18
Posterior arch width lower jaw (mm)	− 0.23	0.76
Anterior arch width lower jaw (mm)	− 0.48	1.35
Intercanine distance upper jaw (mm)	0.61	1.52
Intercanine distance lower jaw (mm)	− 0.14	0.52

### Cephalometric results

#### Skeletal analysis

The examined patients showed an average initial *Y*-axis angle of 67.49° ± 3.84°, indicating a tendency towards a vertical growth pattern (reference: 66° ± 1°). The comparison of the cephalometric variables revealed no significant changes in the sagittal position of the maxilla and the mandible (Table 6). The NS-Gn angle was also not significantly affected by the CMA. However, the ANB angle, the Wits appraisal, and the individual ANB angle were significantly reduced after CMA (ANB: − 0.71 ± 0.77°; *p* = 0.0035; Wits: − 1.99 ± 1.74 mm; *p* = 0.0003; individual ANB: − 0.33 ±0.59°; *p* = 0.0455).

**Table 6:** Cephalometric changes in measurements from T1 to T2

	Initial records (T1)	±	Intermediate records (T2)	±	Difference	±	*p*-value
SNA (°)	81.88	3.94	81.53	4.02	− 0.35	1.11	n. s.
SNB (°)	76.84	4.23	77.21	4.20	0.37	1.14	n. s.
ANB (°)	5.04	1.33	4.33	1.37	− 0.71	0.77	0.0016
Wits (mm)	3.88	2.33	1.89	2.76	− 1.99	1.74	0.0004
indiv. ANB (°)	1.27	1.93	0.94	1.97	− 0.33	0.59	0.0398
NS-Gn (*Y*-axis; °)	67.49	3.84	67.20	4.10	− 0.29	1.45	n. s.
U1 - NS (°)	97.11	6.05	96.19	7.05	− 0.92	3.04	n. s.
U1 - SpE (°)	103.47	6.91	102.40	7.12	− 1.07	2.90	n. s.
L1 - GoMe (°)	96.78	7.12	99.71	6.58	2.94	2.52	0.0003
U6 MB cusp - PTV (mm)	20.36	3.37	18.80	2.88	− 1.56	1.79	0.0034
U6 M cont - PTV (mm)	23.38	3.26	21.71	2.80	− 1.66	1.82	0.0023
U6 - FH (°)	73.69	5.40	68.11	5.38	− 5.58	3.75	< 0.0001
U3 cusp - PTV (mm)	41.78	3.61	39.61	2.95	− 2.17	2.16	0.0011
U3 M cont - PTV (mm)	44.32	3.16	42.93	2.94	− 1.39	1.79	0.0072
U3 - FH (°)	95.83	6.00	91.53	3.64	− 4.29	3.52	0.0002
L6 MB cusp - PTV (mm)	18.82	3.05	20.73	3.32	1.91	1.72	0.0005
L6 M cont - PTV (mm)	20.12	3.02	22.06	3.39	1.94	1.32	< 0.0001

#### Dental analysis

The inclination of the upper incisors was not significantly affected. However, CMA resulted in a significant protrusion of the lower incisors (L1-GoMe: + 2.94 ± 2.52°; *p* = 0.0002). We also observed a significant distalization of the upper canines and first molars (U6 MB cusp - PTV: − 1.56 ± 1.79 mm; *p* = 0.0009; U3 cusp - PTV: − 2.17 ± 2.16 mm; *p* = 0.0008), which was accompanied by a significant tipping (U6 - FH: − 5.58 ± 3.75°; *p* <0.0001; U3 - FH: − 4.29 ± 3.52°; *p* = 0.0002). Finally, the CMA resulted also in a significant mesialization of the lower first molars (L6 MB cusp - PTV: 1.91 ± 1.72 mm; *p* = 0.0001).

#### Comparison with the control group

The results of the comparison with the untreated control group are shown in Table 7.

**Table 7:** Cephalometric changes in measurements—comparison of the CMA group with an untreated age- and gender-matched control group (Denver and Oregon Growth Study)

	T2–T1 CMA	±	T2–T1 control group	±	*p*-value
SNA (°)	− 0.35	1.11	0.27	0.65	n. s.
SNB (°)	0.37	1.14	0.07	0.76	n. s.
ANB (°)	− 0.71	0.77	0.19	0.48	0.0004
Wits (mm)	− 1.99	1.74	1.03	2.37	0.0003
indiv. ANB (°)	− 0.33	0.59	0.01	0.41	n. s.
NS-Gn (*Y*-axis; °)	− 0.29	1.45	0.45	1.52	n. s.
U1 - NS (°)	− 0.92	3.04	0.68	2.69	n. s.
U1 - SpE (°)	− 1.07	2.90	1.06	2.22	0.0345
L1 - GoMe (°)	2.94	2.52	1.25	3.22	n. s.
U6 MB cusp - PTV (mm)	− 1.56	1.79	0.13	1.52	0.0035
U6 M cont - PTV (mm)	− 1.66	1.82	0.12	1.46	0.0021
U6 - FH (°)	− 5.58	3.75	1.46	3.16	< 0.001
U3 cusp - PTV (mm)	− 2.17	2.16	0.81	1.29	< 0.001
U3 M cont - PTV (mm)	− 1.39	1.79	0.76	1.09	0.0002
U3 - FH (°)	− 4.29	3.52	0.72	3.31	0.0004
L6 MB cusp - PTV (mm)	1.91	1.72	0.42	0.91	0.0098
L6 M cont - PTV (mm)	1.94	1.32	0.40	1.28	0.0046
L3 – GoMe (mm)	0.94	1.14	0.56	0.99	n.s.
L6 – GoMe (mm)	1.31	0.84	0.60	0.90	0.0137

To consider the changes caused by growth, the results were compared to an age- and sex-matched untreated control group. The skeletal changes of ANB and Wits of the CMA group (ANB: − 0.71 ± 0.77°; Wits: − 1.91 ± 1.74 mm) were significant compared to the control group (ANB: 0.19 ± 0.48°; Wits: 1.03 ± 2.37 mm). Interestingly, protrusion of the mandibular incisors (2.94 ± 2.52°) was not significant compared to the control group (1.25 ± 3.22°), suggesting that normal growth is also associated with a protrusion of the lower incisors. A significant reduction compared to the control group was shown for the distance of the upper first molar and canine to PTV (U6: − 1.56 ± 1.79mm, U3: -2.17 ± 2.16mm vs. U6: 0. 13 ± 1.52 mm, U3: 0.81 ± 1.29 mm) as well as the angulation of these teeth to FH (significant distal tipping; U6: − 5.58 ± 3.75°, U3: − 4.29 ± 3.52° vs. U6: 1.46 ± 3.16°, U3: 0.72 ± 3.31°). Similarly, there was a significant increase in the distance of the lower first molar to PTV in the CMA group compared to the control group (significant mesialization; 1.91 ± 1.72 mm vs. 0.42 ± 0.91 mm). Vertically, there was a significant extrusion of the lower first molar compared to the control group (1.31 ± 0.84 mm vs. 0.60 ± 0.90 mm).

## Discussion

This study demonstrates that the CMA can effectively correct class II malocclusion within three-quarters of a year to a year. However, it is important to notice that the occlusal correction is not facilitated by a pure distalization, as the former name “Carriere Distalizer” suggested. Instead, there are three-dimensional effects that lead to the correction of the occlusion. Firstly, we noticed that not more than 1 mm distalization of the upper molars (0.96 ± 0.80 mm) can be expected when using the CMA. This means that roughly just a quarter of the class II correction (total 3.45 ± 2.33 mm) was achieved by distalization of the upper molars. Instead, almost three-quarters of the class II correction were achieved by distorotation of the upper molars, mesialization of the lower molars, and skeletal effects (bite position correction). Secondly, it is also important to notice that the distalization of the upper molars is accompanied by distal tipping (− 5.58 ± 3.75°). In fact, our results suggest that the CMA causes more distal tipping than bodily distalization. Thirdly, the mesialization of the lower molars and protrusion of the lower incisors indicate a loss of anchorage in the lower jaw. Finally, our results indicate that the CMA has only a mild effect on the skeletal sagittal jaw relationships when used after the pubertal growth peak (the average age at the start of therapy was 13.9 ± 1.8 years).

The results show a significant reduction of the distance of the upper first molars and canines to PTV (distalization) and a significant distoangulation compared to the control group. In the mandible, there is a significant increase in the distance of the first molar to the PTV (mesialization). The protrusion of the lower incisors is not significant compared to the control group. Obviously, protrusion of the mandibular anterior teeth (possibly as a class II dentoalveolar compensation mechanism) also takes place due to growth. In the vertical dimension, a significant extrusion of the lower first molars is observed due to the vertical component of the class II elastics.

Transverse measurements on the models show almost no changes in anterior and posterior dental arch width and intercanine distance before vs. after CMA.

The distalisation distance is quite small. We were able to show in the study that the effect in the occlusion correction (which actually took place to a greater extent than 0.96 ± 0.8 mm) is hardly a distalisation, but a derotation of U6 and mesialisation of L6. In our opinion, the results are therefore clinically relevant and interesting for the practitioner.

We also observed a headgear effect on the maxillary complex as first described by Pancherz for the Herbst appliance [31]. Whereas we did not detect intrusion of the molars, the extrusive effect in the canine/first premolar region resulted in a clockwise rotation of the occlusal plane.

The average treatment time for distalization of 11.85 ± 4.70 months seems to be rather long as compared to fixed functional appliances. This is probably due to the fact that the use of class II elastics requires patients’ compliance. However, is important to consider that the CMA can be started in the late mixed dentition, which often coincidence with the pubertal growth spurt.

Reflecting on the dental effects of the appliance, it seems mandatory that the use of the appliance should always be based on the strict indication. Class II cases with mesiorotated first upper molars or secondary crowding in the maxilla seem to be suitable in this regard. In contrast, the lower incisors should not be proclined or crowded before therapy, as one can expect that the CMA causes a loss of anchorage in the mandible and a protrusion of the lower incisors. In addition, according to the “Sagittal First” concept of Luis Carrière [19], the appliance should only be used if transversal expansion is not necessary.

Since our study is the first investigation of digital model superimpositions, only the results of the cephalometric superimpositions are comparable to other studies. In this regard, it is important to mention that neither Areepong et al. [26], Wilson et al. [27], nor we were able to prove a pure translational movement of the canines as described by the manufacturer (“distal movement of the canine along the alveolar ridge without tipping” [19]). While Areepong et al. demonstrated a distal tipping of the canines of − 7.44 ± 5.56° [26] and Wilson et al. of 5.17 ± 6.10°/8.74 ± 4.53° [27], our study revealed a tipping of − 4.29 ± 3.52°. The numbers of the distal tipping of the upper first molars in our study (-5.58 ± 3.75°) were also comparable with those of Areepong et al. (− 6.45 ± 4.75°) [26] and Wilson et al. (6.52 ± 3.99°/7.03 ± 3.45°) [27]. Sandifier et al. found numbers which were slightly lower (− 3.7 ± 4.7°) [22].

Moreover, the distalization of the canines (− 2.17 ± 2.16 mm) in our study was also comparable to the numbers of Areepong et al. (− 2.24 ± 1.91 mm) [26]. The same was the case for the distalization of the first molars (our study − 1.56 ± 1.79 mm, Areepong et al. − 1.67 ± 1.56 mm [26], Wilson et al. 1.83 ± 2.11 mm/2.14 ± 1.34 mm [27]). The values measured by Sandifier et al. (− 2.5 ± 2.4 mm) [22] were slightly higher.

The effective mesialization of the lower first molars (1.91 ± 1.72 mm) ranged between the numerical values of Areepong et al. (2.51 ± 1.51 mm) [26], Wilson et al. (1.85 ± 1.88 mm/2.44 ± 2.02 mm) [27], and Sandifier et al. (0.9 ± 2.3 mm) [22].

The proclination of the lower incisors caused by the effect of the class II elastics or rather the anchorage loss could also be demonstrated in Yin et al. [23, 25] and Wilson et al. [27]. In our case, the proclination was 2.94 ± 2.52° (L1-GoMe) and is thus somewhat lower than in Yin et al. (L1-NB: 6.1 ± 2.52°) [23] and comparable to the values of Wilson et al. (2.65 ± 3.01°/3.37 ± 2.98°) [27].

The molar correction we found was − 3.5 ± 2.3 mm, which corresponds to the results of Yin et al. (− 3.5 ± 1.7 mm) [23] and is lower compared to the one of Kim-Bermann et al. (− 5.1 ± 2.0 mm) [21].

Similar to Sandifier et al. [22], Kim-Bermann et al. [21], and Wilson et al. [27], a minimal to mild sagittal growth inhibition of the maxilla was observed in our study (SNA: − 0.35 ± 1.11° vs. − 0.2 ± 2.1° [22], − 0.6 ± 1.0° [21], 0.01 ± 1.32°/− 0.30 ± 1.15° [27]). We also found a small anterior development of the mandible (SNB: 0.37 ± 1.14°), which was similar to that observed by Wilson et al. (0.33 ± 1.21°/0.49 ± 1.28°) [27] and Kim-Bermann et al. (0.1 ± 1.0°) [21], but lower than observed by Yin et al. (1.2 ± 1.9°) [23]. The ANB angle decreased by − 0.71 ± 0.77° similar to the one by Kim-Bermann et al. (− 0.8 ± 0.9°) [21] and Wilson et al. (− 0.33 ± 0.92°/0.77 ± 0.75° [27]). During the correction of the skeletal class II, the Wits appraisal also decreased by − 1.99 ± 1.74 mm in our study (Sandifier et al.: − 2.1 ± 3.6 mm [22]; Yin et al.: − 0.5 ± 2.3 mm [23]; Kim-Bermann et al.: − 2.1 ± 2.0 mm [21]). The results of our cephalometric investigations are therefore in accordance with previous ones [21–23, 25–27] and show little to no skeletal effect. However, it is important to realize that we observed a discrepancy between the results of the cephalometric analysis and those of the model superimposition. In fact, the measured distalization of the upper first molars in the cephalometric superimposition was greater than that of the model superimposition (cephalometric X-ray: − 1.56 ± 1.79 mm; model: − 0.96 ± 0.80 mm). This can be explained by the fact that the cephalometric X-rays show skeletal and dentoalveolar changes based on the distance measurement to the pterygoid vertical. Thus, in the maxilla, the growth inhibition of the maxilla (orthopedic effect of − 0.35±1.11 mm) is added to the distalization distance of the first molar (orthodontic effect). In contrast, the model superimpositions only show dentoalveolar changes with the reference of the palatal rugae. Furthermore, the calculated tooth midpoint is used as a reference point in the model analysis, whereas the mesial contact point or cusp tip is used as a reference point in the cephalometric analysis. Moreover, it is also important that the Kircelli analysis [30] measures the distalization distance parallel to the Frankfort horizontal plane. In vertical growth patterns, the distalization direction in the maxilla or the mesialization direction in the mandible is not nearly parallel to the Frankfort horizontal plane.

Before the option of superimposing 3D scans, it was common to use photocopies of the models [30, 32]. Three-dimensional analysis in cone beam computerized tomography represents an alternative to cephalometric and model evaluation, but we have concerns using this method due to radiation exposure [26]. Moreover, these investigations could only be carried out using an auxiliary plane, since the Frankfort horizontal plane was not available in the cone beam computerized tomographies. Possibly dental magnetic resonance imaging, which is currently under development, might be a might be a solution in the future [33].

Interestingly, our results of the CMA combined with class II elastics show similar numbers as when using class II elastics (with fixed appliances) alone [18, 23]. In contrast to the sole application of class II elastics, the joint (ball and socket design) of the CMA allows distorotation of the upper first molar. This is important, since approximately 90% of all class II division 1 cases have rotated upper first molars [34].

An important issue is whether the changes due to CMA (with class II elastics) would also occur equally when using class II elastics on an edgewise appliance.

The effect of class II elastics is well studied in the literature (e.g., in a systematic review by Janson et al. [18]).

Both our CMA results and the class II elastics results [18] show retrusion and extrusion of the maxillary incisors, protrusion of the mandibular incisors, and mesialization and extrusion of the mandibular molars. Molar correction was 3.0 mm for class II elastics [18] and 3.45 mm for CMA comparable.

While the position of the upper first molars did not change in class II elastics studies [18], our CMA study showed a slight distalisation (U6 MB cusp - PTV: − 1.56 ± 1.79 mm) and especially a distal tipping (U6 - FH: − 5.58 ± 3.75 mm) of the upper first molar. In addition, our model evaluation and the study of Yin et al. 2019 were able to demonstrate a derotation of the upper first molar, which cannot take place when using class II elastics on a multibracket appliance, as the upper first molars are anchored in the steel archwire as well [23]. The main difference of CMA is a derotation of the upper first molar caused by the construction of the appliance (ball-and-socket design). An unfavorable side effect of this can be in rare cases a crossbite in the molar region.

Yin et al. (2019) compared CMA with class II elastics [23]. In their study time of class II correction for CMA was significantly shorter than that for class II elastics (CMA: 6.3 ± 2.2 months vs. class II elastics: 10.3 ± 3.9 months).

If the treatment goal is a translational distalization of the upper first molars (and side effects in the lower jaw due to mesialization of the lower first molars are undesirable), it seems advisable to use other devices such as the distal slider or the classical [35] or bone-anchored [30] pendulum. The Beneslider distalization distances of 4.6 ± 1.5 mm with a minimal tipping (1.9 ± 1.3°) can be realized [16]. Similar results can be achieved using the pendulum appliance (distalization: 3.85 ± 1.24 mm; tipping: 4.65 ± 3.45°) [36]. To derotate the first molars, it is, of cause, also an option to use a quadhelix or a transpalatal arch.

In summary, our results indicate that the effects of the CMA are comparable to those of other fixed functional appliances such as the Forsus [5, 6, 37] or Herbst [35, 38]. In fact, it is well acknowledged that these appliances may positively affect the growth of the maxilla and mandible in adolescent patients but have, besides the skeletal effects, also dentoalveolar side effects. Franchi et al. showed that the ANB angle was reduced by − 1.9 ± 1.2° (non-treated control group: − 0.2 ± 0.8°) by the Forsus appliance, but mesialization of the lower first molars (2.4 ± 1.6 mm) and protrusion of the mandibular incisors (6.1 ± 6.3°) occurred [5]. It is therefore important to mention that the greatest skeletal effect can be achieved with the use of classical functional orthodontic appliances during the pubertal growth peak [39, 40], whereby a large part of the class II correction is still dentoalveolar [41].

Our study has some limitations. First of all, the retrospective study design is a major limitation of our study, as some relevant information was not collected. In a prospective study design, it would have been possible, for example, to collect information such as the compliance of the patients (duration of the daily wear of the elastics). Another limitation is that we included both canine to molar CMA (standard) as well as first bicuspid to molar CMA (shorty) to increase the sample size. This is also the case in another study [26] which deals with this topic. Wilson et al. showed that the shorty CMA achieved class II correction similarly to the standard CMA. Only less change in overjet and distal tipping movement of the maxillary canines could be proved [27].

For a three-dimensional analysis of the skeletal effects cone beam, computerized tomography images would have been necessary. For reasons of radiation hygiene, we only performed a two-dimensional skeletal analysis using cephalometric X-rays.

Another limitation is the use of cephalometric X-rays with the double contours caused by the imaging technique.

Another limitation is the rather small number of cases (*n* = 16) and the inclusion of males and females in our study. Future studies should therefore include investigations with larger sample sizes. An interesting and future-oriented option is here the superimposition of models or intraoral scans with cephalometric X-rays or 3D data from cone beam CT in order to comprehensively and precisely investigate dental and skeletal effects at the same time.

## Conclusion

The CMA provides an efficient way of (dentoalveolar) correction of class II malocclusion within the “Sagittal First” concept. However, it is not superior to other appliances (e.g., Headgear, Distalslider, Pendulum, and fixed functional appliances). In the sagittal plane, there is only a slight distalization of the upper first molar. In fact, the main dental effect of the CMA is a distorotation and distal tipping of the upper first molars. Further effects are due to the class II elastics extrusion of the upper canines: a mesialization of the first lower molars as well as a slight correction of the skeletal class II.

Since the distalization of U3/U6 as well as the extrusion of U3/U4 result in a bite opening and a clockwise rotation of the occlusal plane, the appliance is more suitable for patients with a horizontal or neutral growth pattern than for patients with a vertical growth pattern. Despite the use of an Essix retainer, we found a protrusion of the mandibular incisors. It seems mandatory to take these three-dimensional effects into consideration when using the appliance.
